# Enzymes in “Green” Synthetic Chemistry: Laccase and Lipase

**DOI:** 10.3390/molecules29050989

**Published:** 2024-02-24

**Authors:** Dieter M. Scheibel, Ioan Pavel Ivanov Gitsov, Ivan Gitsov

**Affiliations:** 1 Department of Chemistry, State University of New York-ESF, Syracuse, NY 13210, USA; dieter.scheibel@gmail.com; 2 Science and Technology, Medtronic Incorporated, 710 Medtronic Parkway, Minneapolis, MN 55432, USA; ioanpavel.i.gitsov@medtronic.com; 3 The Michael M. Szwarc Polymer Research Institute, Syracuse, NY 13210, USA; 4 Biomedical and Chemical Engineering Department, Syracuse University, Syracuse, NY 13210, USA; 5 BioInspired Institute, Syracuse, NY 13210, USA

**Keywords:** “green” chemistry, enzymes, immobilization, laccase, lipase, wine making, polyphenols, polymerization, linear-dendritic copolymers

## Abstract

Enzymes play an important role in numerous natural processes and are increasingly being utilized as environmentally friendly substitutes and alternatives to many common catalysts. Their essential advantages are high catalytic efficiency, substrate specificity, minimal formation of byproducts, and low energy demand. All of these benefits make enzymes highly desirable targets of academic research and industrial development. This review has the modest aim of briefly overviewing the classification, mechanism of action, basic kinetics and reaction condition effects that are common across all six enzyme classes. Special attention is devoted to immobilization strategies as the main tools to improve the resistance to environmental stress factors (temperature, pH and solvents) and prolong the catalytic lifecycle of these biocatalysts. The advantages and drawbacks of methods such as macromolecular crosslinking, solid scaffold carriers, entrapment, and surface modification (covalent and physical) are discussed and illustrated using numerous examples. Among the hundreds and possibly thousands of known and recently discovered enzymes, hydrolases and oxidoreductases are distinguished by their relative availability, stability, and wide use in synthetic applications, which include pharmaceutics, food and beverage treatments, environmental clean-up, and polymerizations. Two representatives of those groups—laccase (an oxidoreductase) and lipase (a hydrolase)—are discussed at length, including their structure, catalytic mechanism, and diverse usage. Objective representation of the current status and emerging trends are provided in the main conclusions.

## 1. Introduction

### 1.1. Enzymes: History and Distinctiveness

The implementation of enzymes in synthetic chemistry is often viewed as very lucrative due to their high catalytic activity, substrate specificity, stereo- and regio-specificity, and lack of byproducts [1,2]. Although enzyme-mediated processes have been used by mankind over many centuries, the earliest known report of enzyme catalysis is attributed to the French chemist Anselme Payen, who described their catalytic activity in 1833 [3]. The term ‘enzyme’ was coined by Heidelberg University professor Wilhelm Friedrich Kühne as early as in 1877 [4], but the first isolation of an enzyme was accomplished in 1926 by James B. Sumner. He obtained urease in pure crystalline form, work that would later lead to his award of the 1946 Nobel Prize in Chemistry [5]. Since their initial discovery, numerous enzymes have been identified and found to play key roles in almost all metabolic processes in physiological systems. For this reason, they are widely acknowledged as the primary component in the development of biological life due to their ability to facilitate reactions which would otherwise occur too slowly to sustain sufficient metabolic functions. The biological importance of enzymes has generated considerable interest in their study, with a significant focus on the elucidation of their structure and catalytic properties. Detailed knowledge of biologically significant enzymes has perhaps proven most influential in the pharmaceutical and medical fields, where advances in their chemistry has facilitated a better understanding of the mechanism of diseases and the development of more effective treatments [6,7].

The study of enzyme mechanisms has allowed for their general classification into six groups with respect to their catalytic function, as shown in Table 1. As shown, enzymes have been found to catalyze a large breadth of reactions, including industrially useful oxidations of alcohols and amines, hydrolysis and synthesis of esters, and hydrolysis of ethers, among many others [8].

Although incredibly diverse in function and structure, all enzymes have a commonality in possessing a polymer backbone consisting of α-amino acid sequences connected via amide linkages. Furthermore, all enzymes have unique pH and temperature sensitive secondary and tertiary structures resulting from hydrogen bonding between the various amino acid moieties, changes which can significantly affect the catalytic activity [9]. In addition to their high catalytic activity, enzymes are also well known for their substrate specificity, stereo- and regio-specificity, and catalytic efficiency in biological conditions (i.e., at neutral pH and near ambient temperatures). These advantageous characteristics enable them to efficiently catalyze reactions while minimizing organic solvent use and energy expenditure, thus making these biocatalysts highly desirable as alternatives to traditional catalysts. Due to their universal importance, enzymes and their structures, functions, and applications have been the subject of numerous research articles and reviews. More than 120 reviews appeared in January 2024 alone, and their number will certainly reach a thousand by the end of the year. What distinguishes this publication from all others is the humble attempt to assemble in a single text the interrelation between the enzyme mechanism of action, effects of immobilization on catalytic activity, and suitability for “green” chemistry (Section 1), and then direct the readers’ focus on two enzymes from EC1 and EC3 classes—laccase and lipase. They are more selectively discussed in Section 2 and Section 3. While many of their past and more recent applications are briefly mentioned, more detailed analysis is focused on unprecedented and unique reactions and manipulation strategies that have escaped prior attention or are recently emerging.

#### 1.1.1. Enzyme Catalytic Mechanism

As biocatalysts, enzymes function by decreasing the free energy of the activation of the reactions they catalyze. Additionally, as the degradation of enzymes within one cycle is very small, they are able to work over numerous reaction cycles. Although this catalytic ability is due to a variety of factors, enzyme catalysis is primarily attributed to the ability to facilitate changes in substrate orientation, conformation, or polarization due to the enzyme acting as either a proton donor, proton acceptor, or Lewis acid. Alternatively, the enzyme may briefly covalently bind with the substrate, forming a more reactive intermediate [9] (pp. 70–71).

Enzyme catalysis is known to occur at the enzyme active site, a small crevice at which substrates attach to the enzyme via relatively weak intermolecular forces and ultimately undergo chemical transformation. The specific orientation in which two or more substrates position themselves at the active site is recognized as an important factor in the catalytic mechanism. This is due to the enzyme facilitating a more favorable interaction alignment between multiple substrates, replicating the effect of the increased substrate concentration. In a similar manner, enzyme–substrate complexes have also been shown to induce conformational changes to the enzyme–substrate complex, generating a more reactive substrate resulting from increased bond strain or restriction in bond rotation [9] (pp. 69–70).

Like many conventional catalysts, enzymes may also operate by acting as proton donors or acceptors via easily ionizable pendant groups such as histidine, aspartic acid, and lysine. Additionally, enzymes containing metal ions carrying a positive charge, such as zinc or copper, may serve as Lewis acids by accommodating electrons from the substrate [9] (pp. 71–74). All three of these catalytic mechanisms commonly function by activating either an electrophilic or nucleophilic substrate or by stabilizing leaving groups in the reaction. Due to the positive charge of metal ions, metal-containing enzymes may additionally serve to stabilize negative charges of reaction intermediates if a close ion pair could be formed.

High substrate specificity, characteristic of enzymatic catalysis, is often described as a “lock and key” mechanism and has resulted in enzymes being highly sought after in organic synthesis. This specificity is a result of an active site consisting of well-defined, yet flexible, three-dimensional structures stabilized by significant hydrogen bonding of both amide linkages of the polymer backbone and the amino acid side chains. Induced fit theory, first introduced by Daniel Koshland in 1958 [10], proposes that once the substrate attaches to the active site, the produced enzyme–substrate complex is altered in shape by positioning the amino acid side chains of the active site around the substrate, generating the active enzyme–substrate complex. Substrate specificity is therefore determined primarily by the size, shape, and flexibility of the enzyme active site, although specificity may be broadened using cofactors.

#### 1.1.2. Enzyme Kinetics

**Scheme 1:**
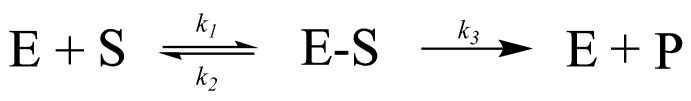
Synthesis of product P through formation of enzyme–substrate complex ES from enzyme E and substrate S.

The chemical kinetics of an enzyme-catalyzed reaction are most often described using the Michaelis–Menten model, first proposed in 1913 [11]. In this model, the enzyme € and substrate (S) reversibly bind with the rate constant *k*_1_, forming an enzyme–substrate complex (ES) (Scheme 1). The formed complex can then either unbind (rate constant *k*_2_) or progress further in the reaction (rate constant *k*_3_), producing the product (P) and free enzyme, which may further react with an additional substrate.

After more than a century, this remarkably simple equation still correctly quantifies enzyme-mediated reactions with few minor adjustments [11]. In the Michaelis–Menten model, the catalytic rate is found to follow first order kinetics, with a linear increase in the catalytic rate with the increasing substrate concentration. However, at high substrate concentrations, the reaction begins to follow zero order kinetics, with the catalytic rate being independent of the substrate concentration. This marked change is due to substrate saturation at the enzyme active site. The rate at which catalysis occurs at a high substrate concentration (i.e., all enzyme active sites have bound a substrate) is referred to as the maximal velocity (V_max_). Another important descriptive parameter is the Michaelis constant K_M_, defined as the substrate concentration at which the reaction rate value is half of the total reaction rate V. They all can be combined in an equation with known (measurable) variables (Equation (1)). It is obvious that V is reversely proportional to K_M_. A detailed discussion and derivation of this equation can be found in the specialized literature. It is briefly discussed here as it provides a facile means for the quantification of enzyme catalytic activity and comparison between catalysts.

(1) $$V=V_{Max}\frac{\left[S\right]}{{\left[S\right]+K}_{M}}$$

#### 1.1.3. Influence of Additives on Enzyme Catalysis

Although many enzymes act independently as catalysts, numerous cases have been observed where they were reliant on the presence of chemical additives known as cofactors. Sometimes referred to as coenzymes or mediators, cofactors may be defined as molecules composed of either a metal ion or organic molecules. They are extraordinarily diverse in structure. As there is relatively limited functional group diversity in the amino acid side chain found on enzymes, an attribute which limits their catalytic versatility, cofactors are most frequently found to provide further chemical reactivity due to the additional functionalities they contain. For this reason, cofactors perform a variety of roles in enzymatic catalysis: an increase in substrate specificity, transport of electrons, atoms, or functional groups; formation of more reactive intermediates; or function as proton donors, acceptors, or Lewis acids [9] (pp. 13–14). Specific compounds acting as cofactors/mediators will be discussed later in this review.

#### 1.1.4. Current Enzyme Usage in Synthetic Chemistry

For the previously discussed reasons (high catalytic activity, substrate specificity, stereo- and regio-specificity, lack of byproducts, sustainability, and stability at mild conditions), it is not surprising that the use of enzymes in synthetic chemistry has increased significantly over the past 50 years [12]. Due to many enzymes tolerating both aqueous and organic environments, they have found use in numerous applications on both the industrial and lab scale [13,14]. Enzymes not inherently compatible with organic solvents may be immobilized, which increases the enzyme stability, a topic discussed in greater detail in Section 1.2. The immobilization of enzymes facilitates the use of enzymatic catalysis with non-polar substrates and additionally suppresses water-driven side reactions, dramatically increasing their versatility in synthetic chemistry. Among many others, enzymes have found uses in synthetic chemistry as biocatalytic alternatives of existing synthetic procedures [15], chiral resolution of racemic substrates [16], oxidation catalysts [17], and as catalysts for carbon–carbon bond formation reactions [18]. Due to society’s extensive use of polymer materials, specific emphasis has been placed on the development of enzymes as environmentally friendly polymerization catalysts. The use of biocatalysts in the synthesis of polymers can be traced to nature, where a myriad of enzymatically produced polymers have been documented. Among them are polysaccharides such as cellulose [19], chitin [20], and glycogen [21], proteins such as collagen, antibodies, and enzymes [22], and polyphenols such as lignin [23]. Enzymes are increasingly considered as viable “green” catalysts for the production [24,25] or degradation [26] of polymers previously made using traditional polymerization techniques.

### 1.2. Enzyme Immobilizations

Although the use of enzymes in synthetic chemistry and industrial applications has increased in recent years, the current practice does not reflect their true potential as catalysts. This limited implementation may be attributed to some limitations of enzyme-mediated catalysis, specifically their sensitivity to environmental conditions such as pH, temperature, and intolerance to organic solvents. Efforts to recycle enzymes in their native state have also proven inefficient, often requiring numerous filtrations or ultracentrifugation to recover the enzyme [27]. The difficulties encountered in enzyme recycling are compounded by the high cost of certain enzymes, making their use in industry cost-prohibitive.

**Figure 1:**
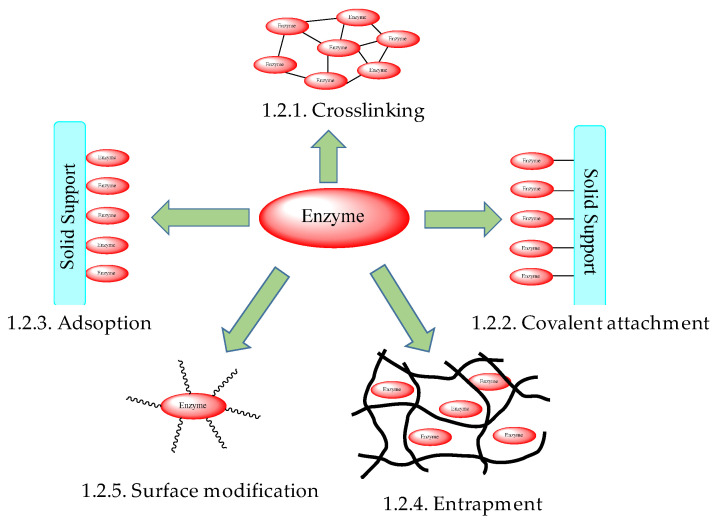
Common methods of enzyme immobilization.

In an attempt to circumvent these unfavorable traits, enzyme immobilization is often employed, whereby enzymes are crosslinked, attached to a support, embedded in a polymeric network, encapsulated, or chemically modified (Figure 1).

Enzyme immobilization primarily serves to increase the enzyme’s thermal and operational stability to pH, organic solvents, and other denaturants, while also assisting in the ease of handling. The improved enzyme stability observed in enzyme immobilization is often attributed to the fixation of the enzyme’s tertiary structure in space via multiple covalent bonds, thereby preventing protein denaturation. Alternatively, immobilization methods such as enzyme encapsulation, entrapment, and chemical modification function by physically separating the enzyme from the reaction media, significantly increasing the enzyme tolerance to pH and organic solvents [28,29]. Of equal importance is the generation of heterogeneous catalysts produced using many fixation techniques, allowing for the simple separation of the immobilized enzyme from the reaction media. This option enables both the easy recycling of the biocatalyst and decreased cost and energy expenditure in reaction workups [27,30]. Enzyme immobilization techniques have also been reported to increase enzymatic activity compared to the native original by means other than preventing protein denaturation. This increase is primarily accomplished using hydrophobic supports which act to sequester hydrophobic substrates in aqueous media [31,32] or by fixing the enzyme into a more catalytically active conformation utilizing site specific covalent binding [33]. In a similar manner, enhanced and/or decreased enzyme selectivity was also observed with immobilization techniques which distort the enzyme active site, a common occurrence with some immobilized enzymes [34]. 

Overall, enzyme immobilization methods should not negatively affect the enzyme activity, allow for efficient substrate and product diffusion, and not obstruct access to the active site. Additionally, immobilization supports should be non-reactive and stable to the reaction conditions and optimally allow for the facile recycling of the scaffold and immobilized catalyst. The aforementioned attributes make enzyme immobilization highly desirable in both industry and academic labs for their cost-saving and catalytic benefits.

#### 1.2.1. Crosslinking of Enzymes

Crosslinking of enzymes to form enzyme aggregates (CLEAs) is the most inexpensive and convenient enzyme immobilization method [35]. CLEAs are most often formed using bifunctional crosslinkers, such as glutaraldehyde, which react with free amino groups in the enzyme (e.g., lysine residues). The significantly reduced cost of covalently induced enzyme aggregation can primarily be attributed to the avoidance of using solid supports, which are often costly and less economically feasible on an industrial scale. By eliminating the solid support, enzyme crosslinking also circumvents activity dilution. As solid supports can comprise up to 99% of the enzyme immobilization system by weight, the enzyme activity, with respect to weight, is significantly reduced, which is an additional drawback. However, CLEAs suffer from several limitations, including limited substrate diffusion and increased enzyme rigidity. Additionally, difficulties arise when attempting to optimally orient the enzyme so as not to occlude the active site. Overall, while increased enzyme stability is often accomplished, a marked decrease in activity is nearly universally observed [27,36].

#### 1.2.2. Covalent Attachment of Enzymes to Solid Scaffolds

**Figure 2:**
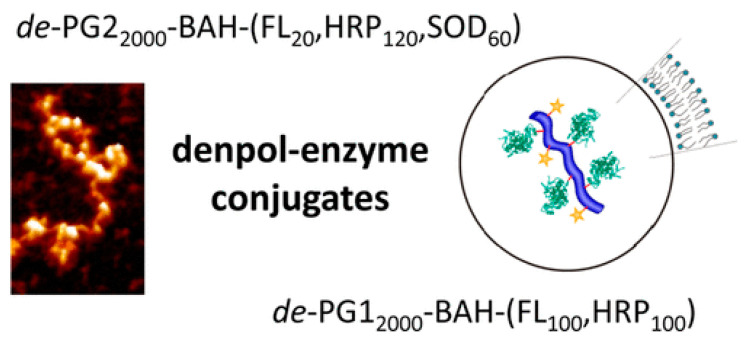
Atomic force microscopy image (**left**) and schematic structure (**right**) of fluorescently labeled polycationic, dendronized polymer (denpol) carrying horseradish peroxidase (HRP), and superoxide dismutase (SOD) attached to the backbone by bis-aryl hydrazone (BAH) bonds. Reproduced with permission from [42]. Copyright (2013) American Chemical Society.

The use of solid supports in enzyme immobilization aims to retain the favorable increased environmental stability and reusability of enzyme immobilization while mitigating the unfavorable qualities of enzyme crosslinking, namely poor substrate diffusion and low activity retention. A myriad of supports have thus far been investigated, including inorganic metal oxide supports—titanium oxide [37], aluminum oxide [38], and zirconium oxide [39], synthetic polymers—poly(styrene) [40], poly(vinyl alcohol) [41]—and even dendronized polymers have served as carriers of enzymatic function [42], as shown in Figure 2.

Biopolymers—chitosan [43], collagen [44], and cellulose [45]—and magnetic particles [46] have also been used. Halloysite nanotubes/polymer combinations, often used in protective coatings [47,48], have been increasingly evaluated as solid enzyme scaffolds [49,50]. The covalent immobilization of enzymes to solid supports is most favorable for costly enzymes due to the stability and enzyme retention it facilitates. It allows for recycling the enzyme with minimal loss in catalytic activity over subsequent reaction cycles. However, as the enzyme binding is nonreversible, the cost of use is increased by the inability to reuse the solid support. As with enzyme crosslinking, covalent binding to supports also causes a habitual decrease in enzymatic activity, resulting from restricted enzyme mobility [27]. Due to the numerous functional groups located on the enzyme pendent moieties, a variety of coupling methods have been developed for the attachment of the biocatalysts on solid supports. Most often utilized in the covalent coupling are the amino acid repeat units lysine, cysteine, tyrosine, and both glutamic acid and aspartic acid bearing amine, sulfide, phenol, and carboxylic functional groups, respectively. As amines can often be deprotonated without causing irreversible enzyme denaturation, they provide a facile means of coupling enzymes to scaffolds with surface functional groups such as epoxy [51], aldehyde [52], isothiocyanate [53], and activated ester [54]. In contrast to epoxy- and aldehyde-based coupling techniques, which are simpler and compatible with a broader range of buffers, activated esters and isothiocyanates undergo facile hydrolysis in the presence of water, leading to low enzyme immobilization or necessitating a large excess of activating agent [27].

Carboxylic groups of enzymes may similarly be used for covalent attachment to solid supports. Activating agents such as 1-ethyl-3-(3-dimethylaminopropyl)carbodiimide (EDC) are often used to generate activated esters, which can react with supports functionalized with nucleophilic functional groups (amines, sulfides, and alcohols) [55]. However, as previously mentioned, the high rate of hydrolysis of activated esters necessitates the use of a large molar excess. Furthermore, activating reagents such as EDC are not site-specific and can induce enzyme crosslinking by coupling with amines of other enzymes, significantly decreasing the catalytic activity. The use of EDC is therefore primarily limited to the formation of enzyme aggregates [56].

The high nucleophilic character of thiols at physiological pH has also made them attractive linking agents in enzyme immobilization. The efficiency of thiol-based coupling reactions at neutral pH facilitates the formation of enzyme-support catalysts with minimal loss of catalytic activity due to pH-induced denaturation [57]. Cysteine residues of enzymes are most commonly used in thiol-based coupling reactions with solid supports bearing thiols, epoxides, or activated ester functional groups for the formation of disulfides, thioethers, and thioesters, respectively. However, more stable and selective binding may be conducted using thiol-ene “click” chemistry via coupling with unsaturated carbonyls like maleimide derivatives [58] or through the formation of strong gold-sulfur bonds, which have demonstrated the ability to improve enzyme activity and thermal and pH stability [59]. 

Tyrosine moieties in the enzyme backbones may additionally be used in the covalent immobilization of enzymes. Attachment via tyrosine residues is most advantageous due to the relatively limited occurrence of tyrosine repeat units on the enzyme exterior, which facilitates attachment at specific sites [60]. Of additional benefit is the widespread use of enzymes to facilitate tyrosine-based enzyme coupling, specifically by the oxidoreductases horseradish peroxidase, HRP [61], laccase [62] and tyrosinase [63]. Both HRP and laccase are most often utilized for the formation of enzyme aggregates [62] due to their ability to rapidly oxidize phenols, generating phenoxy radicals which undergo radical coupling to form biphenyl or phenyl ether linkages. In contrast to the aforementioned phenolic coupling, tyrosinase can be employed to oxidize tyrosine residues to their catechol derivative, and subsequently to the reactive o-quinone. The generation of the o-quinone facilitates simple coupling to thiols and amines in either enzymes or solid supports.

#### 1.2.3. Adsorption of Enzymes onto Solid Supports

**Figure 3:**
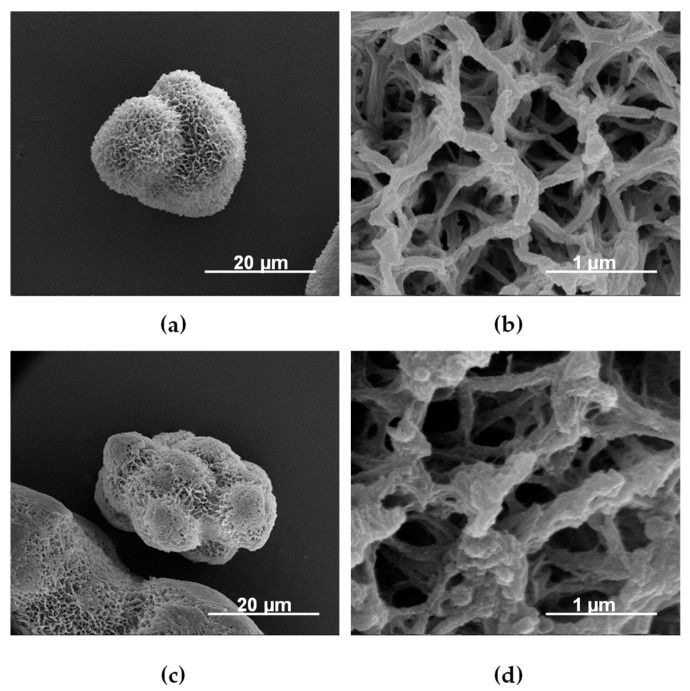
Scanning electron microscopy images of poly(amide 6) microparticles with Fe core before (**a**,**b**) and after (**c**,**d**) laccase adsorption. Modified from the original [68]. This work is licensed under CC BY-NC-ND 4.0.

The first physical immobilization of an enzyme on a solid substrate was reported as early as in 1916, when Nelson and Griffin published their results on the activity of invertase adsorbed on charcoal and aluminum hydroxide [64]. Since then, several strategies have emerged where enzymes may be adsorbed onto solid supports through van der Waals forces, hydrophobic interactions, hydrogen bonding, or ionic bonding [27,65]. Unlike covalent attachment, physical absorption is reversible, allowing for the reuse of the enzyme support upon the depletion of catalytic activity through the absorption of the new enzyme. Additionally, as the enzyme may desorb and reabsorb, enzyme mobility is minimally restricted, allowing for significant retention of catalytic activity with respect to the native enzyme. This approach has proven particularly useful for the immobilization of enzymes on metal-organic frameworks [66,67]. Poly(amide)-coated magnetic microparticles were successfully employed as easily recoverable scaffolds for enzyme-mediated organic reactions and possible environmental cleanup, as shown in Figure 3 [68].

Other polymer-coated magnetic particles have also been reported as potential solid supports of enzyme function [69]. However, these methods are heavily dependent on the isoelectric point of the adsorbed enzyme and universally suffer from sensitivity to environmental conditions such as pH, ionic strength, and temperature. Furthermore, due to the weak forces facilitating enzyme absorption to the support, the immobilized catalyst has limited recyclability, resulting from substantial enzyme leaching [70]. Ionic adsorption may also be achieved through the numerous amine and carboxylic groups of the enzyme, which contribute to an overall positive or negative surface charge. The efficacy of ionic adsorption is heavily dependent on both the isoelectric point of the enzyme and the surface charge of the solid support. In a similar manner, hydrophobic amino acid residues (e.g., alanine, valine, and leucine) and large glycosidic domains on the enzyme may afford adsorption to hydrophobic supports [71]. Novozym^®^ 435 could be considered as the classic example of this immobilization strategy [72]. It contains *Candida antarctica* lipase B (CALB) embedded in a macroporous polymer network formed by crosslinked poly(methyl methacrylate) and divinylbenzene. The formulation has found widespread use in industry and academia, with only a few applications quoted herein [73,74,75]. Further examples of this particular approach will be discussed in some detail in the subsequent sections. As both adsorption methods do not involve significant modification of the enzyme, instead utilizing intrinsic properties of the biocatalyst, they are viewed as highly advantageous in commercial applications, owing to their economic feasibility and high activity retention.

#### 1.2.4. Enzyme Entrapment in Polymeric Supports

**Figure 4:**
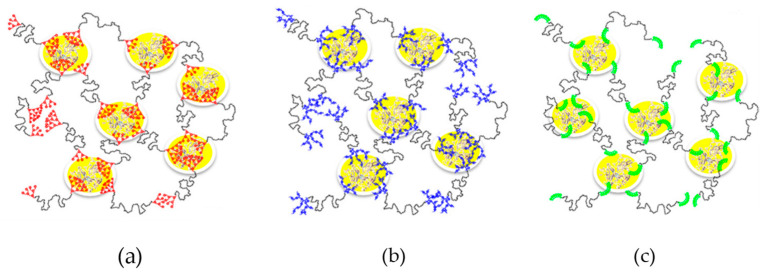
Schematic diagram of the complex formation between the enzyme and linear–dendritic block copolymer (**a**); linear–hyperbranched block copolymer (**b**) and linear–linear block copolymer (**c**). Modified from the original [77]. This work is licensed under CC BY-NC-ND 4.0.

Alternatively, rather than being attached to the surface of solid supports, enzymes may instead be embedded in situ within nanoporous structures formed during a polymerization process [76] or during the spontaneous self-assembly of amphiphilic copolymers [77]. In the last case, an activity enhancement of up to 1248% (!) was observed. It was assumed that the entrapment was facilitated by hydrophobic–hydrophobic interaction between the enzyme and the complimentary blocks of the copolymers, as was postulated in the previous section, and as shown in Figure 4. One of the distinct advantages of this strategy is that the entrapped enzyme remains relatively mobile, facilitating, in this way, unhindered access to the active site.

Applications of preformed hydrogels [78] or inorganic frameworks [79] for this purpose have also been reported. The entrapment of enzymes within such a matrix primarily serves to increase the mechanical stability and does not afford significant advantages to environmental tolerances. As the enzyme is physically immobilized and has minimal interaction with the matrix, which could otherwise cause denaturation and decreased enzyme mobility, entrapment methods can allow for the significant retention of enzyme activity. However, such methods often incur problems with activity retention over multiple reaction cycles due to enzyme leaching from the matrix, although this shortcoming can be partially resolved by decreasing the pore size of the matrix. Perhaps the most significant disadvantage of enzyme immobilization via entrapment is the limited substrate diffusion through the matrix to the enzyme active site, a problem compounded by decreasing the matrix pore size in attempts to reduce enzyme leaching [80]. These factors, in addition to the low loading capacity and possible enzyme deactivation during the immobilization procedure, severely limit the industrial usefulness of enzyme entrapment methods.

#### 1.2.5. Enzyme Stabilization in Micelles and by Surface Polymeric Modification

Related to enzyme immobilization is the incorporation of enzymes in micelles or reverse micelles, which can be utilized in reactions using either aqueous or organic solvents, respectively. Surfactants used for the micellar stabilization of enzymes may be composed of small molecules [81] or polymers [82] and be either ionic or non-ionic in nature. In both aqueous and organic conditions, micelles primarily serve to assist in the dispersion of enzymes, preventing aggregation and precipitation [83]. Furthermore, the reverse micelles used in organic media prevent enzyme denaturation by isolating the enzyme from the organic solvent, an attribute which has significantly increased their use in catalyzed reactions which are often inhibited by water and necessitate anhydrous conditions [84,85]. Like enzyme entrapment, the stabilization of enzymes within micelles has a negligible restriction on the enzyme mobility, thus increasing the retained activity. However, micelle stabilization has not proven suitable for many enzymes as the use of surfactants, specifically anionic surfactants, has habitually caused enzyme denaturation [83] As micelle stabilization does not allow for the reuse of enzymes, surfactants are most suitable for the stabilization of inexpensive enzymes such as lipase [83].

**Figure 5:**
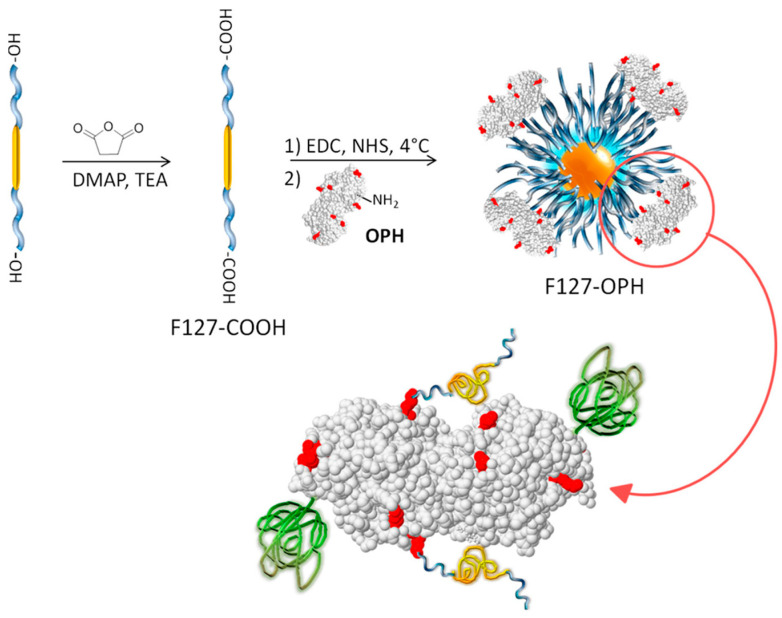
Conjugation of carboxy-terminated Pluronic 127 to organophosphorus hydrolase (OPH) through sequential modification with 1-Ethyl-3-(3-dimethylaminopropyl)carbodiimide (EDC) and N-hydroxysuccinimide (NHS). Reproduced with permission from [91]. Copyright (2014) American Chemical Society.

An alternative method of enzyme stabilization is the chemical modification of enzymes, most often conducted through the covalent attachment of polymers to the enzyme surface [86]. As with the covalent attachment of enzymes to a solid support, chemical modification improves the enzyme rigidity and thermal stability, also concomitantly providing better solubility in organic solvents. While a variety of polymers have been investigated as chemical modifiers for enzymes, including polysaccharides [87], polypeptides [88], and various synthetic vinyl polymers [89], poly(ethylene glycol), PEG, and derivatives are by far the most commonly used [90]. Referred to as PEGylation, the attachment of PEG to enzymes may be conducted using a diverse range of coupling agents, such as activated esters at the polymer chain ends. An example is shown in Figure 5 [91]. 

In selected cases, the successful PEGylation produced conjugates with activities 4 to 3000 (!) times that of the native enzyme [92]. Although some correlations have been found between the polymer molecular mass and degree of attachment (i.e., the number of polymers attached to the enzyme) with enzyme activity, no conclusive trends relating these factors to more than one enzyme has been determined [93]. The inability to forecast what effect polymer attachment has on the enzyme activity and stability, even with structurally similar enzymes, necessitates an individual investigation of each enzyme–polymer composite [88,94] The low predictability and high cost of development has greatly limited the viability of chemical modification as a general method for the improvement of enzymes as catalysts. 

#### 1.2.6. Immobilized Enzymes as Polymerization Catalysts

The use of enzymes for environmentally benign polymerizations was briefly mentioned in Section 1.1.4. A few additional examples will be discussed here. The immobilization of a mutant endoglucanase on gold nanoparticles for the synthesis of cellulose through β-cellobiose polymerization was reported by Nakamura et al. [95]. Although the immobilized enzyme displayed lower activity than its native counterpart, a polymer with a higher degree of crystallinity was obtained. The same group achieved noticeably higher polymerization efficiency and produced cellulose fibrils in contrast to the plate-like crystals synthesized using the native enzyme [96].

The immobilization of oxidoreductases, mainly horseradish peroxidase (HRP), was commonly conducted for the polymerization of phenol [97] and aniline derivatives [98]. The polymerization of aniline for the creation of poly(aniline)-based electrodes was demonstrated by Kausaite-Minkstimiene et al., who immobilized glucose oxidase on carbon rods via glutaraldehyde [99]. Submicrometer-sized vesicles formed from sodium bis(2-ethylhexyl)sulphosuccinate (AOT) facilitated the polymerization of aniline mediated by class III peroxidases [100]. It was found that the addition of amphiphilic linear-dendritic block copolymer improves the operational stability of horseradish peroxidase isoenzyme C, but this effect was not further exploited. The usefulness of immobilized HRP for the polymerization of vinyl monomers was also validated [101]. This was achieved through the immobilization of HRP inside poly(N-isopropyl acrylamide)/chitosan interpenetrating networks, and the resulting complexes were used for the polymerization of acrylamide. The reported HRP system was found to be significantly more stable than its native counterpart while also maintaining polymerization efficiency and providing enzyme recyclability.

The following two sections will discuss two enzymes that are among the most widely used biocatalysts in “green” synthesis.

## 2. Laccase

### 2.1. Laccase Catalysts in Nature

Laccases (benzenediol:oxygen oxidoreductase, EC 1.10.3.2) belong to a family of multicopper core oxidases containing a minimum of four copper atoms at their active site, classified as either Type 1 (T1), Type 2 (T2), or binuclear Type 3 (T3) Cu centers [102]. First discovered in 1883 by Hikorokuro Yoshida as a component of lacquer sap from *Rhus vernicifera* [103], a broad variety of laccases have since been isolated, with particular interest in those derived from fungal sources due to their ease of production, low cost, and high redox potential. Characterized by their broad substrate specificity, laccases are capable of catalyzing one-electron oxidations of a myriad of organic and inorganic substrates, although they are best known for their oxidation of phenols with the concomitant reduction in oxygen. 

Being found in fungi, plants, and microorganisms, laccase has many roles in nature (Table 2). It is perhaps most well-known for its function in numerous fungi, where it catalyzes the degradation of lignin and humic acid, most probably employing naturally occurring radical mediators such as syringaldehyde and vanillin [104]. Laccase has also been suspected to play a major role in the synthesis of melanin-like pigments, where phenol and catechol derivatives are oxidized to strongly absorbing quinones [105]. Similar to fungal sources, laccases found in bacteria serve to catalyze the reactions involved in the degradation of lignin and other phenols, in addition to the synthesis of pigments. In bacteria, however, laccase also plays the unique role of assisting in the resistance to spores and pathogenesis, most likely by catalyzing the oxidation of toxic compounds [106]. 

In distinction to both fungal and bacterial sources, laccase in plants has been found to play a primary role in lignin synthesis by catalyzing the polymerization of monolignols accounting for the lignin component of plant cell walls, and is therefore of interest for the synthesis of synthetic lignin and lignin-like polymers [107]. Analogous to plants, insects utilize laccase as a catalyst in the development of their cuticle. This process occurs through the laccase oxidation of catechols to their corresponding quinones which can crosslink proteins of the cuticle, allowing for its hardening [108].

**Table 2:** Laccases: sources, molecular mass, and catalytic function in nature.

Source		Molecular Mass (kDa)	Natural Function	Reference
Fungal	*Scytalidium thermophilum*	82	Lignin degradation Pigment formation	[109]
	*Rhizoctonia solani*	70–85	Lignin degradation Pigment formation	[109]
	*Trametes versicolor*	58	Lignin degradation Pigment formation	[110]
Bacterial	*Strepotomyces antibioticus*	67.5	Phenoxazinone synthesis	[111]
	*Bacillus subtilis*	65	Spores pigmentation UV and H_2_O_2_ resistance	[112]
	*Pseudomonas syringae*	72	Copper resistance	[113]
Plant	*Acer pseudoplatanus*	59.9	Lignin Synthesis	[114]
	*Malus domestica*	74	Lignin Synthesis	[115]
	*Rhus vernicifera*	110	Lignin Synthesis	[116]
Insect	*Manducta sexta*	220–280	Cuticle Scleritization	[117]
	*Sarcophaga bullata*	90–100	Cuticle Scleritization	[118]
	*Periplaneta americana*	185	Cuticle Scleritization	[119]

### 2.2. Laccase Structure and Catalytic Pathway

**Figure 6:**
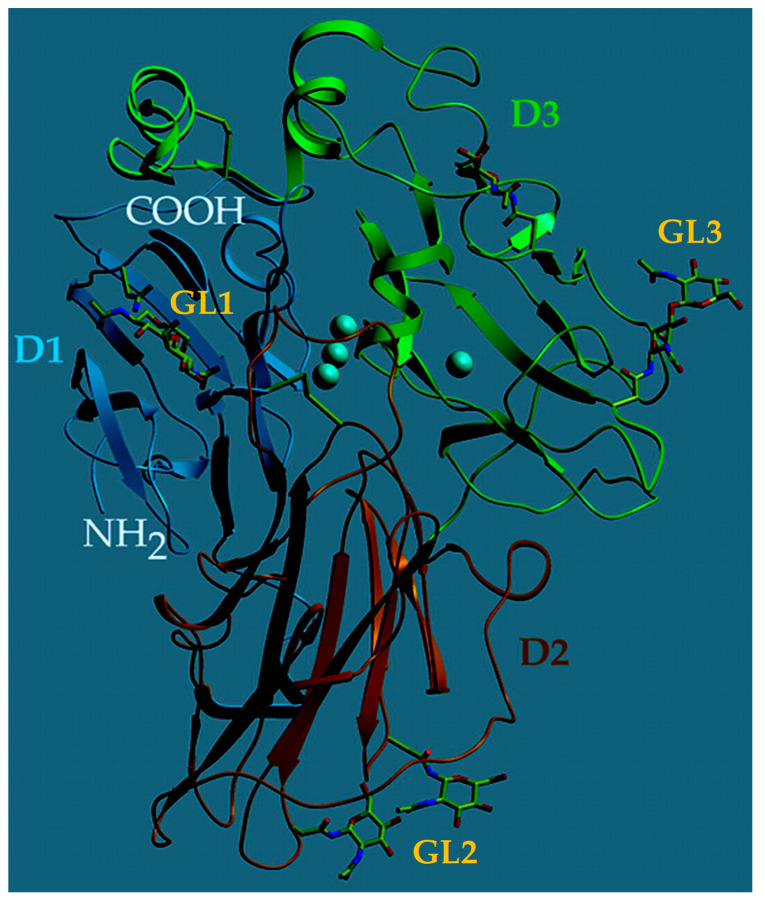
Three-dimensional structure of *Trametes versicolor* laccase. The protein domains (D1–3) are shown in dark blue, red, and green; the four Cu atoms are depicted in light blue and the glycoside anchoring sites (GL1–3) are marked in yellow. Modified from [120]. This work is licensed under CC BY.

Although heavily dependent on the source, laccases most commonly possess a molecular mass of 60–70 kDa and can be divided into three nearly homogenous domains of β–barrel type structures which surround the active site at the center of the enzyme (D1–3, Figure 6) [120]. Laccases are further composed of covalently linked glycosidic regions, constituting between 10–45% of the enzyme’s mass (GL1–3, Figure 6) [120]. These glycosidic regions serve to protect the enzyme from thermal inactivation, proteolytic attack, and solvent-induced denaturation [121]. The substrate binding pocket in the interior of laccase is surrounded by hydrophobic fragments and histidine/aspartic acid/glutamic acid residue located nearby. This particular residue participates in hydrogen bonding with substrates containing –OH or –NH_2_ groups, assisting in deprotonation [122]. This enzyme structure provides the optimal catalytic activity of laccase between 50–70 °C and an optimal pH varying greatly with the substrate [123]. 

**Scheme 2:**
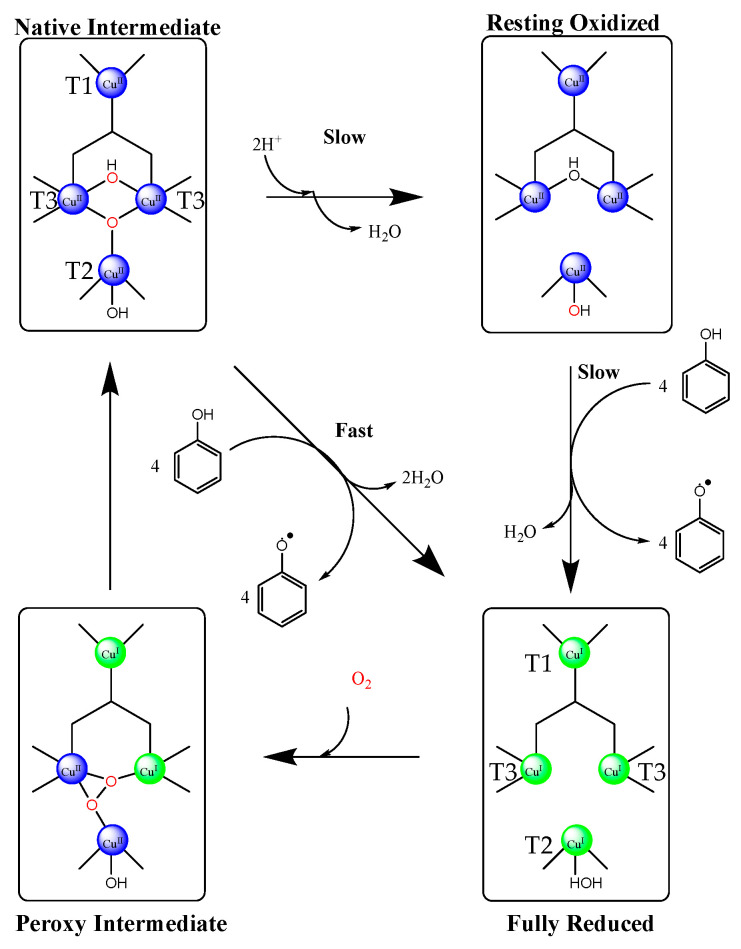
Laccase catalytic cycle depicting the native intermediate (fully reduced oxygen), the oxidized resting state, the fully reduced form (after substrate oxidation), and the peroxy intermediate (partial oxygen reduction). Phenol is used as a model substrate. Cu^II^—blue, Cu^I^—green. Modified from the original [125]. Copyright (2010) American Chemical Society.

The catalytic mechanism of laccase has been thoroughly investigated through electron paramagnetic resonance spectroscopy and X-ray crystallography, allowing for the near complete elucidation of the enzyme active site function [124]. Of primary importance for understanding the laccase mechanism of action is the elucidation of the role T1, T2, and binuclear T3 copper centers play in the process. It is schematically presented in Scheme 2 [124].

Investigation of the active site has revealed that the T1 site is ligated by two nitrogen atoms in histidine moieties and a thiolate residue on the enzyme. This site serves as a long-range intramolecular electron shuttle, directing electrons from the substrate to the T2 and T3 copper atoms, and is therefore the position of substrate oxidation. The two copper atoms of the T3 site are ligated by three histidine groups each and are bridged by a hydroxide anion. The T2 site is ligated by an additional two histidine residues and a water molecule. Catalysis is initiated through the oxidation of the substrate at the T1 copper site via one electron transfer. Substrates containing either hydroxyl or amine groups are often activated by the enzyme through deprotonated aspartic acid groups neighboring the active site, decreasing the strength of O–H or N–H bonds, and consequently facilitating oxidation. Electrons are then shuttled from the T1 copper to the T2 and T3 atoms, where molecular oxygen is bound and able to accept the transported electrons. Altogether, four single electron transfers are conducted to reduce one molecule of oxygen. The complete catalytic cycle is illustrated in Scheme 2. The redox potential of this cycle is heavily dependent on the laccase source, but is most often between 440–790 mV.

The K_M_ values are also source-dependent and vary between 11 and 20 μmol.

#### 2.2.1. Radical Mediators in Laccase Catalysis

**Figure 7:**
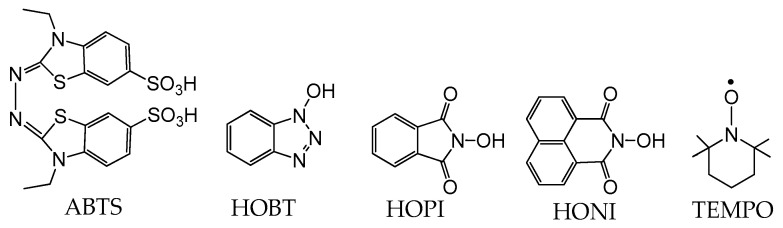
Selected mediators. 2,2′-azino-bis(3-ethylbenzothiazoline-6-sulfonic acid), ABTS; 1-Hydroxybenzotriazole, HOBT; N-hydroxyphthalimide, HOPI; N-hydroxynaphthalimide, HONI and 2,2,6,6-tetramethylpiperidine-N-oxyl, TEMPO.

**Scheme 3:**
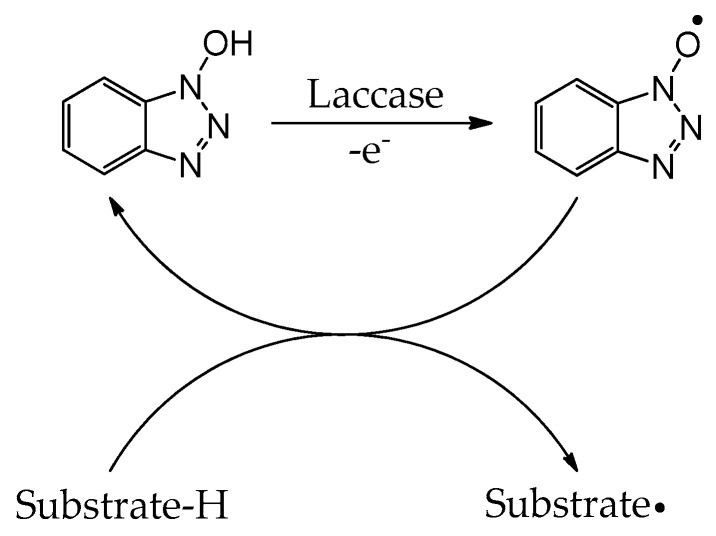
Oxidation of HOBT through laccase and electron transfer to substrate with concomitant regeneration of the nitroxide radical mediator.

While laccases have an inherently broad substrate specificity, the array of substrates capable of undergoing induced oxidation is greatly increased by the addition of catalytic amounts of radical mediators [126]. Such mediators include 2,2′-azino-bis(3-ethylbenzothiazoline-6-sulfonic acid) (ABTS), 2,2,6,6-tetramethyl-1-piperidinyloxy (TEMPO), 1-hydroxybenzotriazole (HOBT), N-hydroxyphthalimide (Figure 7), and other nitroxide-based mediators [126]. The electron-rich naphthalimide (HONI) has also been successfully employed for the enhanced oxidation of benzo-α-pyrene in water mediated by *Trametes versicolor* laccase regio-electively modified with amphiphilic linear-dendritic block copolymers [127]. The notable advantage of this enzyme–copolymer complex was that both the mediator and the polyaromatic hydrocarbon are highly hydrophobic but could selectively be bound by the dendritic blocks in the vicinity of the enzyme active site and facilitate/undergo oxidation. Nitroxide-based radical mediators most often function as electron shuttles and are primarily applied in reactions where the substrate is either incapable of fitting into the enzyme active site or unable to reach the active site due to spatial limitations in substrate diffusion. The electron shuttle mechanism by which these mediators act is widely accepted to occur via the laccase oxidation of the mediator’s N-OH group, producing nitroxide radical N–O● (Scheme 3). The resultant radical migrates from the enzyme active site to the substrate, where it can abstract a hydrogen radical, oxidizing the substrate and regenerating the mediator [126].

**Scheme 4:**
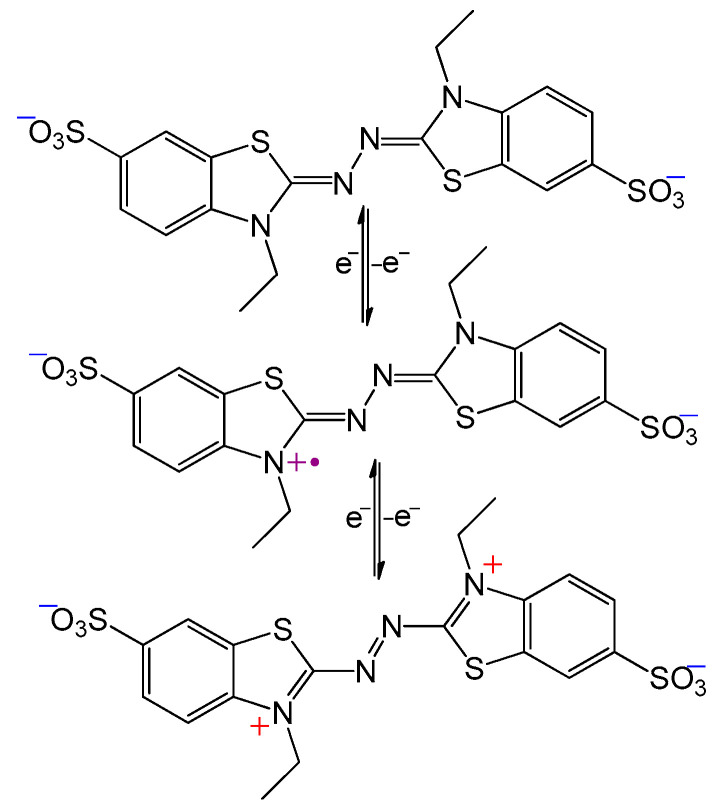
Oxidation of ABTS dianion (**top**) to the radical cation (**middle**), and finally to the dication (**bottom**).

Alternatively, radical mediators may function as generators of oxidative species of larger redox potential, thereby increasing the choice of substrates. An often-used mediator which utilizes this mechanism is ABTS (Scheme 4). During the initial oxidation of ABTS (E° = 690 mV) by laccase (E° = 440–790 mV), the radical cation ABTS^●+^ is produced. Due to the inherent instability of the radical-cation, it can spontaneously and reversibly form the dication ABTS^++^ (E° = 1100 mV), a strong oxidant capable of oxidizing many non-phenolic compounds [35].

### 2.3. Laccase Catalysis: Commercial Applications

The success of laccases as biocatalysts in industrial applications is due primarily to their wide substrate specificity, offering great catalytic adaptability. This versatility facilitated laccase usage in the pulp and paper industry, biofuel production, food industry, manufacturing of textiles, and synthesis of small molecules, among others [128]. Mimicking their natural function in fungi, laccases are most often utilized in the pulp and paper industry as a catalyst to facilitate the degradation of lignin in paper [128,129]. The removal of lignin, which imparts a yellow color, allows for the brightening of the paper, with an identical method available for the bleaching of cotton. The use of laccase as a bleaching agent serves to replace the historically used chlorine-containing reagents, which have proven to be environmental pollutants and deleterious to ecological health. Biofuel production also utilizes laccase’s ability to degrade lignin in biomass, which would otherwise inhibit the efficient hydrolysis of polysaccharides to produce ethanol [130]. Laccase use in the food industry is predominantly involved in the oxidation and removal of undesirable phenols in beverages via radical coupling polymerization. The removal of these phenols in beverages such as wines, beers, and juices often provides stabilization, clarification, and flavor enhancement [131]. 

Winemaking is one area that has exploited the beneficial activity of laccase through purification through the removal of substances prone to oxidative transformation—antho-cyanins, coumaric acids, and flavans. It can also be used for process monitoring and control by biosensing, as well as in the creation of a special class of wines known as Noble Rot wines. The initial identification of oxidative enzymes (i.e., polyphenol oxidase and laccase) in the wine industry resulted from research on brown pigmentation in white grape must and juice. This reaction occurs due to oxidizing dihydroxy phenolic compounds to quinones, which, upon further oxidation, turn to brown pigments [132,133]. The first historical use of laccase focused on the removal of the phenolic content to elucidate the stabilization of wine. Using laccase isolated from *Trametes versicolor*, Brenna and Bianchi achieved a reduction in catechin with a model wine solution in the presence of O_2_ excess [134]. Browning at 420 nm increased with each successive treatment, further supporting what Macheix et al. suggested in 1991 [132]. The study also mentioned experiments carried out on Riesling grape must; however, no results were reported regarding the effectiveness of the laccase bioreactor in removing the phenolic content directly from grape must. In a more advanced study, Minussi et al. used capillary zone electrophoresis and the kinetics of phenol removal to monitor the total phenolic content, total antioxidant potential, and polyphenols to determine the efficacy in using *Trametes versicolor* laccase to treat grape must [135]. Phenol degradation by laccase occurred very quickly for catechins, but slowly for stilbenes and benzoic/cinnamic acid derivatives. Furthermore, in red musts, the treatment primarily showed reductions in the must antioxidant properties, while white musts showed higher reductions in the total phenols rather than total antioxidant potential. Therefore, it was concluded that laccase as a method for stabilizing wines is more feasible in white grape musts, until selectivity studies for laccase from *Trametes versicolor* are conducted. In a 2013 study, the laccase-catalyzed oxidation of gallic acid was compared to the chemically catalyzed procedure, and it was found that oxidative kinetics increased 3-fold in the presence of laccase [136]. Furthermore, the laccase released from *Botrytis cinerea*, responsible for bunch rot in wine grapes, lead to a significant decrease in the phenolic content. Though not often desirable due to its impact on wine color, there is a strong case for using laccase to reduce the total phenolic content in wines.

The initial method for measuring the polyphenol content in wine involved the use of the Folin-Ciocalteu reagent and following the reaction spectrophotometrically [137]. To improve upon this process, Gamella et al. suggested the electrochemical estimation of the polyphenol index in wine using a laccase biosensor [138]. Using *Trametes versicolor* laccase, they found significant differences between the laccase immobilized on a glassy carbon electrode and the Folin-Ciocalteu method. Furthermore, both methods had different specificities towards different phenolic compounds based on chemical structures. Numerous other studies, however, showed comparable results to the Folin-Ciocalteu method, including immobilized enzymes (laccase from *Trametes versicolor* and tyrosinase from mushroom) on graphite screen-printed electrodes modified with ferrocene [139], on a platinum-silver electrode base sensor [140], and laccase from *Trametes versicolor* and *Trametes hirsuta* using screen-printed electrodes of multi-walled carbon nanotubes as sensors [141]. In a later study, another group used laccase purified from *Ganoderma* sp. *Rckk02* immobilized on silver/zinc oxide nanoparticles and electrochemically deposited onto a gold electrode. The oxidation potential was determined using guaiacol and a good correlation was found between the spectrophotometric method and this one. Furthermore, the biosensor was found to have retained 75% of its activity after 200 uses over 5 months [142]. 

Aside from improving wines through post-fermentation clarification, the fungus *Botrytis cinerea* can also produce a special class of wines prior to grape harvest. The production of these sweet botrytized wines is credited as originating in the Tokaj region of Hungary [143] and Schloss Johanisberg region of Germany [144], followed by the Sauternes region in France [145]. Today, these wines are also produced in regions of the United States, Australia, New Zealand, and South Africa, where favorable conditions for noble rot exist [145]. The development of desirable fungus infections, known as noble rot, occur under ideal climatic and soil conditions—notably, constant water availability and in soils poor in nutrients [146]. These conditions most readily occur due to the stimulation of infection from nocturnal humidity, morning dew, or fog that occurs in valleys or areas bordering bodies of water [147]. Grapes impacted by noble rot exhibit the withering of the fruit that is characteristic of overripening, and as such, contain a higher concentration of sugar and various aromatic hydrocarbons that constitute the unique aromas present in noble rot wines. The process by which grapes achieve these desirable attributes is due to the oxidation of aromatic metabolites through enzymatic reactions from laccase, producing new compounds such as furfural, phenylacetaldehyde, and lactones, amongst others [148].

The textile industry also benefits from the laccase-catalyzed oxidation of colorless phenols and anilines (e.g., phenylenediamines and aminophenols) for the production of a diverse range of dyes [129]. Laccases have additionally been explored as catalysts in peptide synthesis [149], the oxidation of alcohols [150], the generation of iodine from iodide [151], and the synthesis of various anti-cancer drugs [152] and antibiotics [153].

### 2.4. Laccase Use in Bioremediation Applications

**Figure 8:**
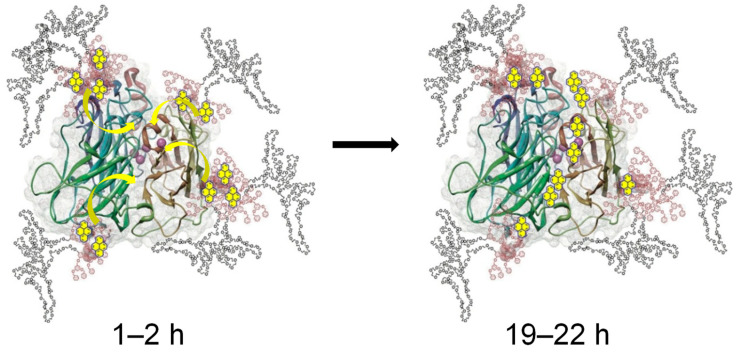
Substrate harvesting and migration to laccase active site, as revealed through spectrophotometric investigation of pyrene fluorescence [157].

The implementation of oxidoreductases for use in bioremediation applications has developed into an expansive sub discipline of biocatalysis. Laccase has specifically shown significant promise as a means of treating industrial effluents, which often contain a myriad of hazardous chemicals. Laccase has thus far demonstrated the ability to effectively oxidize a variety of compounds found in wastewaters, such as halogenated phenols [154,155] and polyaromatic hydrocarbons [127,156]. The unprecedented oxidation of fullerene C_60_ in water was also achieved through a laccase/linear-dendritic copolymer complex at close-to-ambient temperatures [157]. It was made possible by the activity enhancing effect of the linear-dendritic copolymer used—native laccase K_M_ = 26.2 μmol; V_max_ = 72.5 nmol·s^−1^·mL^−1^ vs. laccase–polymer complex values of 15.8 (K_M_) and 101.2 (V_max_), respectively [156]. This study is unique as it was able to trace for the first time a migration of potential substrates from the enveloping polymer shell to the active site. It was achieved by monitoring the fluorescence of pyrene, an environment-sensitive fluorophore that cannot be chemically transformed by the enzyme. It was established that pyrene was almost instantaneously sequestered into the hydrophobic dendritic pockets anchored at the surface of the enzyme and slowly migrated to the active site over a time span of more than 15 h (Figure 8), an important finding that was used in subsequent “green” chemistry reactions with the same polymer–enzyme complex.

**Scheme 5:**
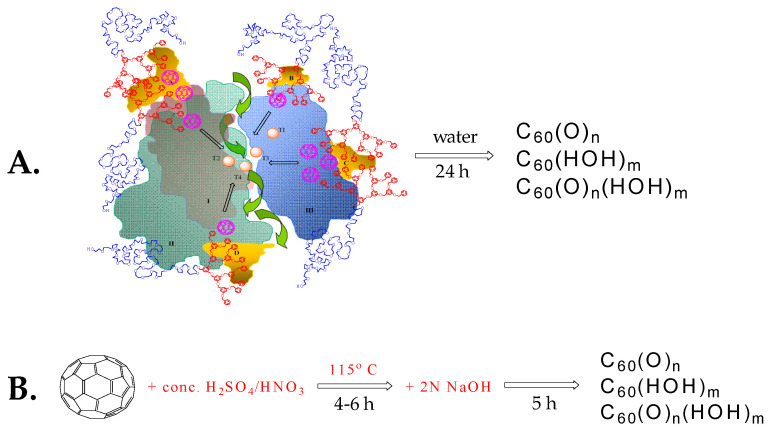
Oxidation of fullerene C_60_. (**A**). Process mediated by laccase/linear-dendritic complex [157], green arrows indicate oxygen entry to and products exit from the enzyme active site. (**B**) Process catalyzed through sequential treatment with concentrated H_2_SO_4_, concentrated HNO_3_, and 2N NaOH [158].

The environmental benefits of this method compared to previously published procedures [158] were clearly distinguishable (Scheme 5); nonetheless, the editors of *Angewandte Chemie*, *The Journal of the American Chemical Society*, *Journal of Molecular Catalysis B: Enzymatic*, and *Green Chemistry* did not have the scientific courage to publish it, despite the solid experimental proof and overly positive reviews.

Laccase is also able to oxidize [159] or polymerize [160] bisphenol A and derivatives, polychlorinated biphenyls [161], dioxins [162], and other phenolic compounds [68], known as mutagens and endocrine disruptors. The removal of these hazardous chemicals by laccase occurs via radical mediated dehalogenation and/or oxidative polymerization, resulting in compounds of markedly decreased toxicity and water solubility, lowering their environmental impact. Furthermore, laccase has been validated to degrade various pharmaceuticals, with particular emphasis on steroids [163], antibiotics [164], and other micropollutants [165].

The decolorization of dyes in industrial effluents has also garnered significant attention. Due to their diverse composition, the degradation of dyes in wastewater treatment necessitates a method capable of degrading a wide range of substrates. The broad substrate specificity of laccase makes it an ideal candidate as a catalyst for the treatment of industrial effluents for the textile industry, where dyes are commonly used. Laccase has demonstrated its viability in this role by successfully oxidizing the most common classes of dyes used in the production of textiles, including azo (comprising mono-, di-, and triazo compounds) [166], triphenylmethanes [167] indigo [168], anthraquinone [169], and xanthene dyes [170]. The addition of radical mediators, such as those discussed in Section 2.2.1, further expand the scope of dyes able to be degraded by laccase and often increase the degradation efficiency [77]. The implementation of laccase as a catalyst in the degradation and decolorization of dyes from industrial effluents therefore serves to improve water quality by decreasing the color, turbidity, and toxicity, while most notably avoiding the production of toxic arylamines.

### 2.5. Laccase-Catalyzed Polymerizations

**Scheme 6:**
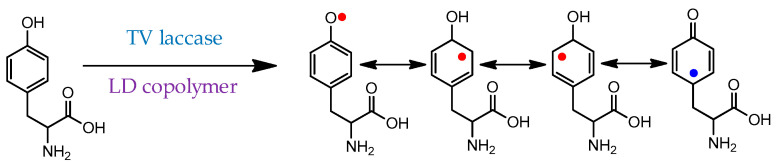
Initial radical formation during the polymerization of tyrosine mediated by laccase/linear-dendritic copolymer complex [171]. Red dots—productive radicals leading to C-C or C-O couplings; blue dot—nonproductive radical.

Mirroring many of their natural functions, laccases have also been thoroughly studied as polymerization catalysts, with many of their substrates being phenol, catechol, and aniline derivatives. An example of a phenolic compound (tyrosine) polymerization mechanism is shown in Scheme 6 [171]. Notably, this substrate is strongly hydrophobic, but the polymerization was accomplished in water facilitated by the same linear-dendritic copolymer complex (see Figure 8 and associated text).

**Scheme 7:**
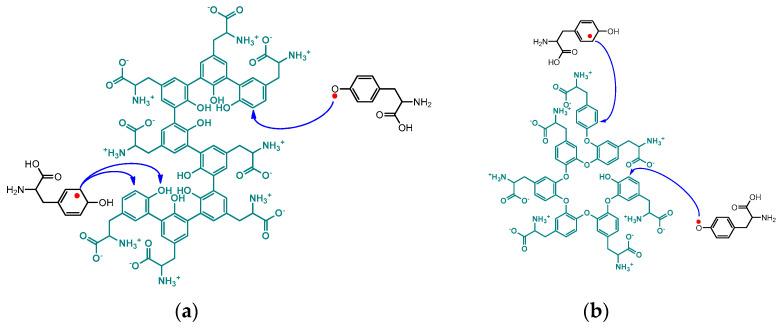
Suggested propagation mechanism through addition of tyrosine monomer radicals to the chain ends of unnatural poly(tyrosine). (**a**) Addition to all C-C coupling chain; (**b**) addition to all C-O coupling chain.

The *oxo*- and *ortho*-centered radicals participate in sequential polyaddition reactions. The resulting polymer behaves as a typical poly-zwitterion, decreasing or increasing in size with changes in the pH of the aqueous medium [171]. This behavior hints at the flexible linear structure of the unnatural poly(tyrosine) chain, thus revealing the propagation mechanism, as shown in Scheme 7.

**Figure 9:**
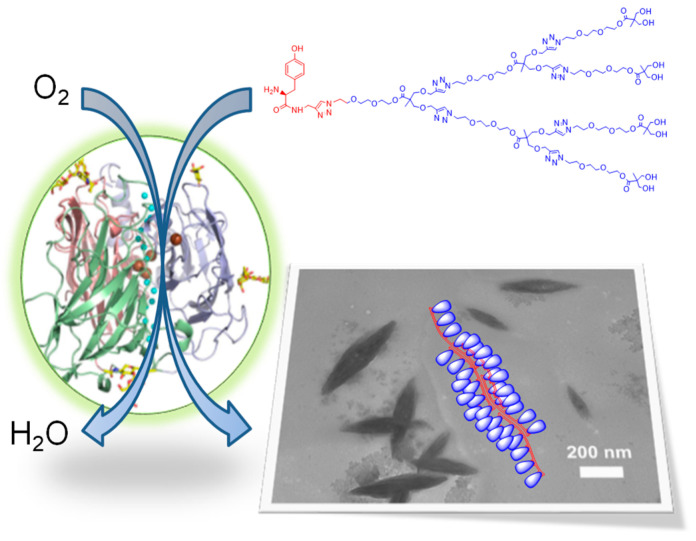
Polymerization of tyrosine-dendron macromonomers mediated by *Trametes versicolor* laccase in aqueous medium. The resulting amphiphilic dendronized polymers spontaneously self-assemble in water, as revealed through transmission electron microscopy (bottom right image). Modified with permission from [172]. Copyright (2021) American Chemical Society.

A similar approach was used for the first-ever enzyme-mediated synthesis of dendronized self-assembling unnatural poly(tyrosine), poly(tyr-Gn) [172]. The number of repeating units that laccase was able to “stitch” in a polymer chain depended on the dendron generation and decreased from 12 in poly(tyr-G1) to 9 for poly(tyr-G2) and 7 in poly(tyr-G3). Nevertheless, all three polymers spontaneously aggregated in aqueous media into unique supramolecular structures, Figure 9. 

**Scheme 8:**
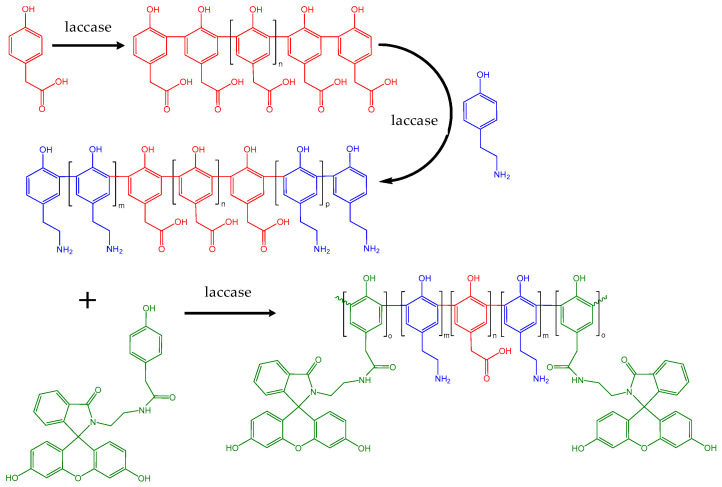
One-pot synthesis of a penta-block copolymer formed through “quasi-living” copolymerization mediated by native or copolymer-complexed laccase from *Trametes versicolor*. Modified with permission from [173]. Copyright (2020) American Chemical Society.

A never-before attempted synthesis of multi-block copolymers by a single enzyme in a one-pot process was also recently accomplished. Laccase catalyzed the first “quasi-living” copolymerization through the sequential addition of multiple naturally derived phenolic comonomers conjugated with a model drug (ibuprofen) or fluorescent groups (fluorescein or rhodamine) [173], as shown in Scheme 8. The multi-block copolymers produced by the last method have low cytotoxicity and can undergo cellular uptake, a good indication for their promising theragnostic potential. The published protocol opens promising pathways for the construction of copolymers with properties tailor-made for specific applications.

There are several reports on the laccase-mediated synthesis of poly(catechol), an electroactive polymer that is often applied as a thin film in biosensing applications [174]. Poly(phenyl ether), a commercially useful polymer due to its optical clarity and high thermal and radiation resistance, has also been produced through an oxidative radical coupling and subsequent decarboxylation of para-hydroxybenzoic acid derivatives [175]. 

The enzymatic synthesis of intrinsically conductive and thermally stable poly(aniline) has perhaps received the most attention. In contrast to conventional methods of poly(aniline) synthesis using horseradish peroxidase [176] or oxidizing reagents [177], the use of laccase does not require stoichiometric amounts of oxidizer or hydrogen peroxide. Laccase therefore provides a safe and inexpensive method of synthesizing poly(aniline), a polymer widely used in energy storage devices, biosensors, and anticorrosion coatings [178,179].

The use of laccase as a polymerization catalyst is not, however, limited to phenolic substrates. Reports of laccase facilitating the polymerization of vinyl monomers are numerous, with high molecular masses and yields often being achieved. Unlike the polymerization of phenolic compounds, which occurs through oxidative radical coupling, in vinyl polymerizations, laccase most often acts as a catalyst for polymerization initiation. Thus, it has been described as a catalytic initiator for the atom radical transfer polymerization of N-vinylimidazole, producing a polymer of low dispersity for use in drug delivery, fuel cell membranes, and polyionic liquids [180]. More traditional laccase-catalyzed free radical polymerizations conducted with acetylacetone as a radical mediator have used a variety of substrates, including acrylamide [181], methyl methacrylate, and styrene, for the synthesis of commercially significant polymers [182]. A limited number of laccase copolymers were formed by either vinyl comonomers [182] or phenolic and vinyl comonomers [183]. Phenolic comonomers were also grafted onto solid supports such as cellulose [184], although these have yet to attain significant commercial value.

## 3. Lipase

### 3.1. Lipase Catalysts in Nature

**Scheme 9:**
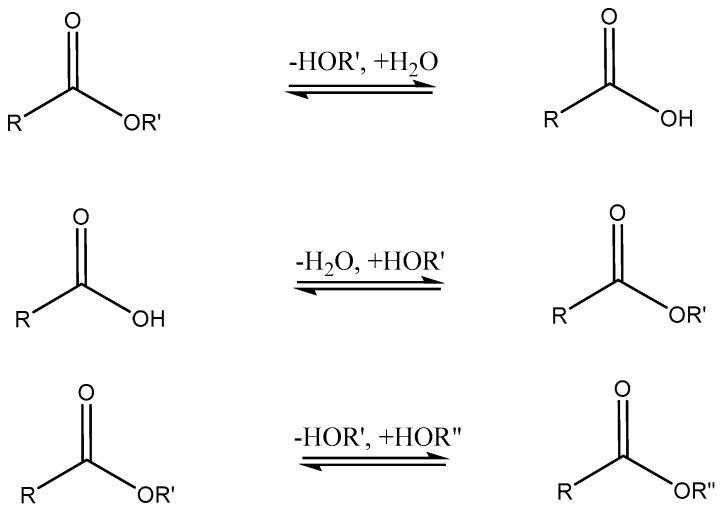
Common reactions mediated by lipases: hydrolysis (**top**), esterification (**middle**), and transesterification (**bottom**).

Lipase (hydrolase, EC 3.1.1.3) is a type of esterase first described by Claude Bernard in 1848 when he observed that pancreatic extracts were capable of emulsifying fats via saponification [185]. Most well known for their ability to catalyze the hydrolysis of triglycerides, lipases are found in nearly all plants, animals, and microbes, where they serve to aid in the synthesis or digestion and metabolism of fats [186]. The three major reactions catalyzed by lipases are shown in Scheme 9.

### 3.2. Lipase Structure and Catalytic Pathway

**Figure 10:**
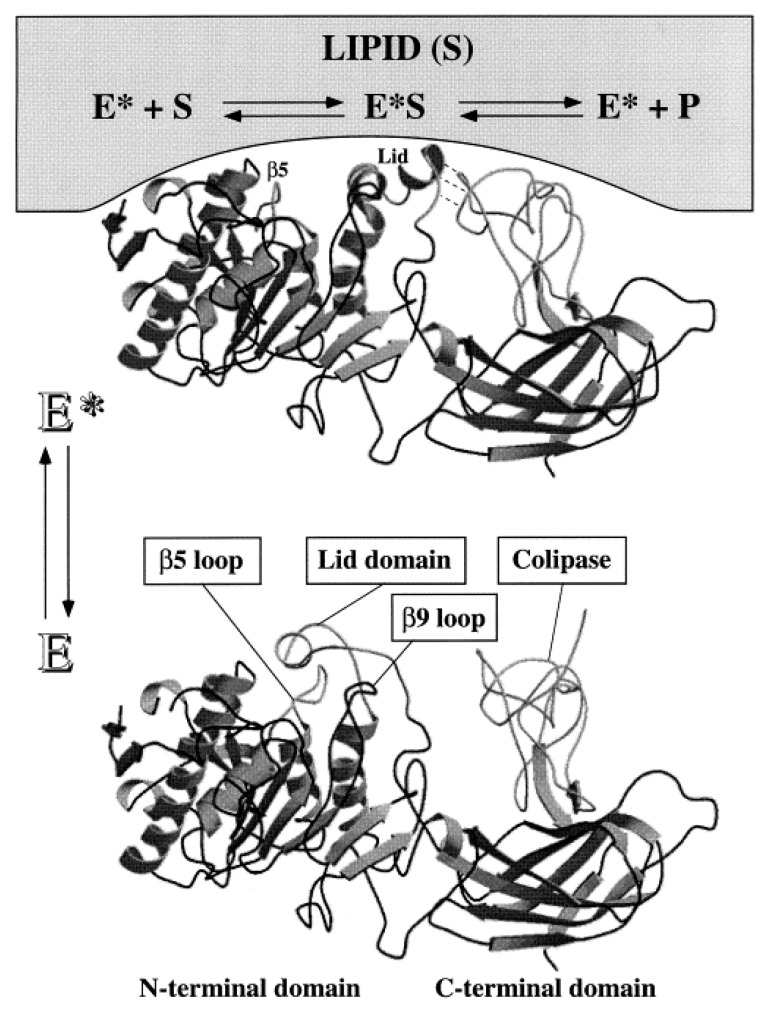
3D structures of human pancreatic lipase. E*—open conformation at the lipid/water interface; E—closed conformation in solution. Reproduced with permission from [189]. Copyright (1998) Elsevier.

Due to their numerous sources, lipases are diverse in structure, with molecular masses ranging between 20–75 kDa [187]. Although the specific structure of lipases varies considerably, the majority contain a mobile amphipathic polypeptide chain, commonly referred to as a “lid” [188]. In aqueous media, the hydrophilic region of the lid faces outwards towards the polar solvent, denoted as a “closed” position, occluding the enzyme active site. Contrarily, organic media facilitates a change in enzyme conformation, whereby the hydrophobic region of the lid faces outwards, in contact with the non-polar solvent, denoted as the “open” position, allowing for substrate binding [189]. The movement of the lipase lid is referred to as “interfacial activation” [190] and is illustrated in Figure 10. 

The presence of this lid causes lipases to operate most efficiently at water–oil interfaces, showing limited activity towards dissolved substrates. For this reason, lipases do not follow typical Michaelis––Menten-type kinetics, instead showing an S-shape activity that is dependent on the substrate concentration [187]. Although dependent on the source, most lipases display optimal activity between pH 4–9 and temperatures between 30–40 °C. Some lipases, however, have demonstrated the ability to remain active up to 90 °C [191]. The active site of lipases has long been known to be composed of histidine, aspartic acid, and serine moieties, collectively referred to as the catalytic triad, which serves as an acid-base catalyst. Mechanistically, lipase acts to generate a reactive enzyme–substrate complex with the substrate (E*S, Figure 10). This complex is connected covalently via an ester bond with the lipase serine residue. Then, E*S can react with nearby nucleophiles such as water (hydrolysis reactions) or alcohols (esterification or transesterification reactions). Due to the role ‘acid-base’ chemistry has in the catalytic mechanism, the dependence of the lipase activity on the pH primarily arises from conditions that enable the deprotonated state of the aspartic acid residue while maintaining the histidine protonation [187].

### 3.3. Lipase Applications in Organic Chemistry

Owing to their relatively high tolerance to organic solvents, lipases have found numerous uses in organic synthesis. Utilizing their native function, they have served as “green chemistry” catalysts for the hydrolysis of triglycerides [192]. Due to their inherently high stereospecificity, the catalytic hydrolysis of chiral esters was used for facile chiral resolution [193]. Lipase has also garnered attention for its ability to catalyze the hydrolysis and transesterification of fatty acids for the production of biofuels [194]. However, recent work has significantly broadened the scope, with reports demonstrating the ability of lipase to catalyze a variety of unnatural reactions, including epoxide formation [195], aldol reactions [196], Michael Additions [197,198], and thiol addition to imines [199]. These newfound catalytic roles of lipases towards unnatural substrates serve to substantially increase their versatility and usefulness as biocatalysts in organic synthesis [200].

### 3.4. Lipase-Catalyzed Polymerizations

**Scheme 10:**
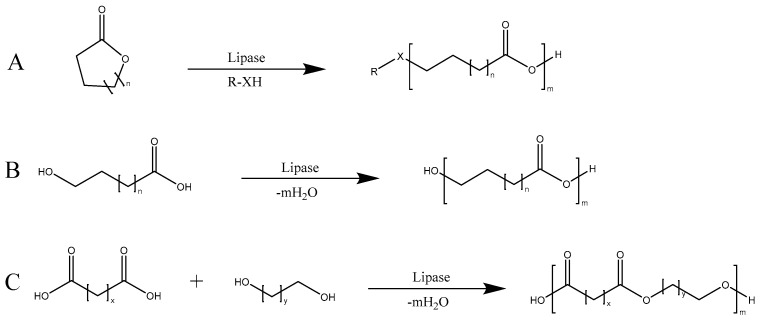
Lipase-mediated polymerization of lactones (**A**), oxyacids (**B**), and dicarboxylic acids and diols (**C**).

In addition to the synthesis of small molecules, lipase is often used as catalyst for the synthesis of polymers. Such polymers include polyesters which may be prepared through the ring-opening polymerization (ROP) of lactones and condensation polymerization of α,ω-hydroxy acids or the polycondensation of dicarboxylic acids and diols, as shown in Scheme 10. 

**Scheme 11:**
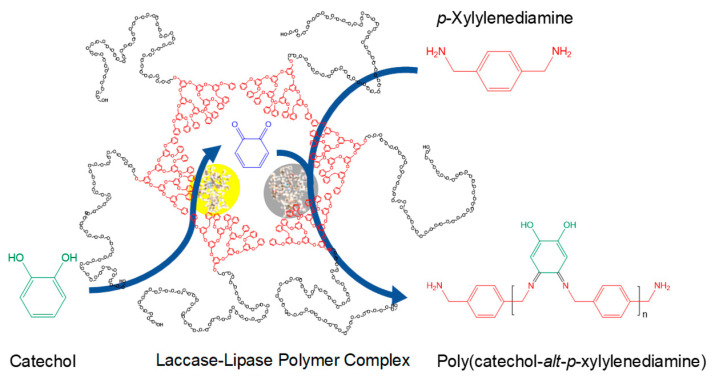
Alternating copolymerization of catechol and m-xylylenediamine mediated by a laccase–lipase pair co-compartmentalized in supramolecular polymer complexes. Reproduced with permission from [207]. Copyright (2019) American Chemical Society.

Of primary interest are the lipases used in ROP applications due to the high molecular mass and yield that may be achieved, a result of the absence of the need for a stoichiometric monomer ratio and lack of water byproduct, which would promote the hydrolysis of the formed polyester [201]. Since that first 1993 article, investigations on the ROP of lactones have since produced numerous types of polymers from a variety of 4–17 membered ring monomers with a diverse range of side chains [202,203,204,205]. Furthermore, due to the high selectivity often observed in lipase catalysis, optically active polymers are capable of being produced from racemic monomer mixtures [206]. Expanding upon the previously published elegant process by Witayakran and Ragauskas [197], a combination of lipase and laccase embedded in amphiphilic supramolecular assemblies enabled the unprecedented formation of perfectly alternating copolymers, as shown in Scheme 11 [207].

In addition to polyesters, lipase has demonstrated the ability to initiate the synthesis of polycarbonates and polyethers through the ROP of cyclic carbonates [25], natural cyclic monomers [208], and oxiranes [209,210].

Lipases have also been utilized for the post-polymerization functionalization of polymers. The functionalization of polymers containing hydroxyl end groups, such as poly(ethylene glycol), have been modified with a variety of acrylates and halogenated acetic acid derivatives, using lipase as a catalyst for the production of block copolymers or for the facile attachment of fluorescent end groups [211]. Modification of the interior of the polymer chain has also been demonstrated through the lipase-catalyzed epoxidation of polybutadiene and the stereospecific esterification of polymers containing either hydroxyl or carboxylic acid functional groups [26]. It should be noted that lipase immobilization on different scaffolds, including poly(styrene), poly(propylene), acrylic resins, and ceramic porous supports, greatly enhances the activity of this biocatalyst in the ROP of lactones [212]. Similar work was conducted by Noda et al., who utilized surfactant-coated lipases to induce the ROP of lactones of various ring sizes in organic media [213]. The surfactant/enzyme complexes were found to display significantly higher polymerization rates and were noted to produce narrower dispersity polymers in higher yields [213].

## 4. Conclusions

This review has shown only a small glimpse into the galaxy of enzymes, their discovery, and characterization. There is an ever-increasing trend of applying their unique properties and catalytic specifics in diverse applications that are both highly efficient and environmentally benign [214]. However, enzyme chemistry has yet to come to full fruition, largely due to the expense of these biocatalysts and the difficulty in recovering and reusing them after each reaction cycle. This is especially true in industrial settings where high activity must be maintained throughout many reaction cycles. An additional area of concern regarding the use of enzymes in synthetic chemistry is their susceptibility to undergoing structural changes when exposed to organic solvents and changes in pH and temperature. These changes often lead to a considerable decrease in or the complete loss of enzymatic activity. To mitigate these unfavorable characteristics, substantial attention has been given to the development of enzyme-immobilizing materials, which serve to anchor or envelop the enzyme and diminish the effect that these environmental factors have on enzymatic activity. Several immobilization methods that have gained popularity include covalent bonding to solid scaffolds [215], ionic bonding to scaffolds with charged surfaces [216], and physical entrapment within a physically or covalently crosslinked network [217]. These immobilization methods, however, are often plagued by a considerable decrease in enzymatic activity, resulting from unfavorable alterations in the enzyme mobility and orientation. Ionic and physical immobilization methods are also inhibited by low enzyme retention after repeated cycles, especially at pH extremes.

Future studies should have the main purpose of finding ways to perform the syntheses in aqueous media. Therefore, they should focus on: (a) The design of alternative immobilizing agents, which should be able to universally complex a variety of enzymes, increasing their activity, compatibility with aqueous solvents, and recyclability; (b) The examination of the effect of the newly developed complexing agents and compare their efficacy with previously developed immobilization strategies; (c) The development of industrially adequate protocols for processes carried out in water and close to ambient temperatures and the innovation of socially important technologies, such as bioremediation and environmental clean-up, multi-stage synthesis of substances of biomedical importance, and multi-block copolymers, to name a few.

## Figures and Tables

**Table 1:** Enzyme classes and their catalytic function.

Enzyme Class	Catalytic Function	Selected Enzymes
Oxidoreductase, EC1	Oxidation-reduction reactions involving intermolecular electron transfer	Laccase Horseradish Peroxidase Glucose Oxidase Cytochrome Oxidase
Transferase, EC2	Transfer of functional groups from donor molecules to acceptor molecules	Butyrate Kinase DNA Methyltransferase Glucosyltransferase Thiaminase
Hydrolase, EC3	Hydrolysis	Amylase Lipase Pepsin Urease
Lyase, EC4	Non-hydrolytic bond cleavage	Adenylate Cyclase Carbonic Anhydrase Tryptophan Synthase
Isomerase, EC5	Molecular isomerization	Photoisomerase Beta-carotene Isomerase Glucose isomerase
Ligase, EC6	Large molecules coupling	Pyruvate Carboxylase DNA Ligase CTP Synthase

## Data Availability

No new data were created or analyzed in this study. Data sharing is not applicable to this article.
